# Necrotizing Fasciitis: A Review of Management Guidelines in a Large Obstetrics and Gynecology Teaching Hospital

**DOI:** 10.1155/S1064744993000055

**Published:** 1993

**Authors:** C. D. Thompson, A. L. Brekken, W. H. Kutteh

**Affiliations:** 1 Department of Obstetrics and Gynecology University of Texas Southwestern Medical Center Dallas TX USA; 2 Reproductive Endocrinology, Dept. of Ob/Gyn 5323 Harry Hines Boulevard Dallas TX 75235-9032 USA

## Abstract

Necrotizing fasciitis is a severe, life-threatening soft tissue infection that results in rapid and progressive destruction of the superficial fascia and subcutaneous tissue. Because of its varied clinical presentation and bacteriological make-up, it has been labelled with many other names such as acute streptococcal gangrene, gangrenous erysipelas, necrotizing erysipelas, hospital gangrene, and acute dermal gangrene. Although described by Hippocrates and Galen, it has received increasing attention in obstetrical and gynecological literature only within the last 20 years. This review includes two recent cases successfully managed at Parkland Memorial Hospital, Dallas, Texas. The first patient was a 50 year old, morbidly obese, diabetic woman who presented with a small, painful lesion on the vulva. After failing triple antibiotic therapy with ampicillin, clindamycin, and gentamicin, the diagnosis of necrotizing fasciitis of the vulva was made, and she was taken to the operating room for extensive excision. She was discharged home on hospital day 29. The second patient was a 65 year old, obese, diabetic woman with risk factors for atherosclerosis who had a wound separation after an abdominal hysterectomy. Two days later a loss of resistance to probing was noted in the subcutaneous tissue. Necrotizing fasciitis was suspected, and she was taken to the operating room for resection. The patient was discharged home on hospital day 27. The mortality rate after diagnosis of necrotizing fasciitis has been reported to be 30% to 60%. We review the literature and outline the guidelines used in a large Ob/Gyn teaching hospital to minimize the adverse outcome. Lectures on soft-tissue infections are included on a regular basis. The high-risk factors of age over 50, diabetes, and atherosclerosis are emphasized. The need for early diagnosis and surgical treatment within 48 hours is stressed, and any suspicious lesions or wound complications are reported to experienced senior house officers and staff. We use two recent cases to highlight the diagnostic clues and management strategies for this often fatal polymicrobial infection.


Infectious Diseases in Obstetrics and Gynecology 1:16–22 (1993)
© 1993 Wiley-Liss, Inc.

### Necrotizing Fasciitis: A Review of Management Guidelines in a Large Obstetrics and Gynecology Teaching Hospital

C.D. Thompson, A.L. Brekken, and W.H. Kutteh

*Department of Obstetrics and Gynecology, University of Texas Southwestern Medical Center, Dallas, TX*

ABSTRACT

Necrotizing fasciitis is a severe, life-threatening soft tissue infection that results in rapid and progressive destruction of the superficial fascia and subcutaneous tissue. Because of its varied clinical presentation and bacteriological make-up, it has been labelled with many other names such as acute streptococcal gangrene, gangrenous erysipelas, necrotizing erysipelas, hospital gangrene, and acute dermal gangrene. Although described by Hippocrates and Galen, it has received increasing attention in obstetrical and gynecological literature only within the last 20 years. This review includes two recent cases successfully managed at Parkland Memorial Hospital, Dallas, Texas. The first patient was a 50 year old, morbidly obese, diabetic woman who presented with a small, painful lesion on the vulva. After failing triple antibiotic therapy with ampicillin, clindamycin, and gentamicin, the diagnosis of necrotizing fasciitis of the vulva was made, and she was taken to the operating room for extensive excision. She was discharged home on hospital day 29. The second patient was a 65 year old, obese, diabetic woman with risk factors for atherosclerosis who had a wound separation after an abdominal hysterectomy. Two days later a loss of resistance to probing was noted in the subcutaneous tissue. Necrotizing fasciitis was suspected, and she was taken to the operating room for resection. The patient was discharged home on hospital day 27. The mortality rate after diagnosis of necrotizing fasciitis has been reported to be 30% to 60%. We review the literature and outline the guidelines used in a large Ob/Gyn teaching hospital to minimize the adverse outcome. Lectures on soft-tissue infections are included on a regular basis. The high-risk factors of age over 50, diabetes, and atherosclerosis are emphasized. The need for early diagnosis and surgical treatment within 48 hours is stressed, and any suspicious lesions or wound complications are reported to experienced senior house officers and staff. We use two recent cases to highlight the diagnostic clues and management strategies for this often fatal polymicrobial infection. © 1993 Wiley-Liss, Inc.

KEY WORDS

Soft tissue infections, gynecologic infections, obstetrical infections

### CASE REPORTS

### Case 1

A 50 year old white woman ($G_2$,$P_2$) originally presented to the Parkland Memorial Hospital Ob/Gyn Emergency Room complaining of a painful "boil on my vagina." She had a 1 cm follicular abscess on the left vulva at the intertriginous fold that would rub against her thigh as she walked. She denied fever, chills, or other systemic symptoms. Her past medical history was significant for a total abdominal hysterectomy with bilateral salpingo-oophorectomy and appendectomy in 1963, a ventral hernia repair in 1970, and a 20-year history of adult onset diabetes mellitus controlled with glyburide 7.5 mg daily. Her serum glucose was 128 mg/dl. She had a 22 pack/year history of smoking and denied the use of alcohol. Physical examination revealed a morbidly obese (327 pounds) woman who was generally healthy, other than her present-

Address correspondence/reprint requests to Dr. William H. Kutteh, Reproductive Endocrinology, Dept. of Ob/Gyn, 5323 Harry Hines Boulevard, Dallas, TX 75235-9032.

Received July 10, 1992
Accepted December 15, 1992

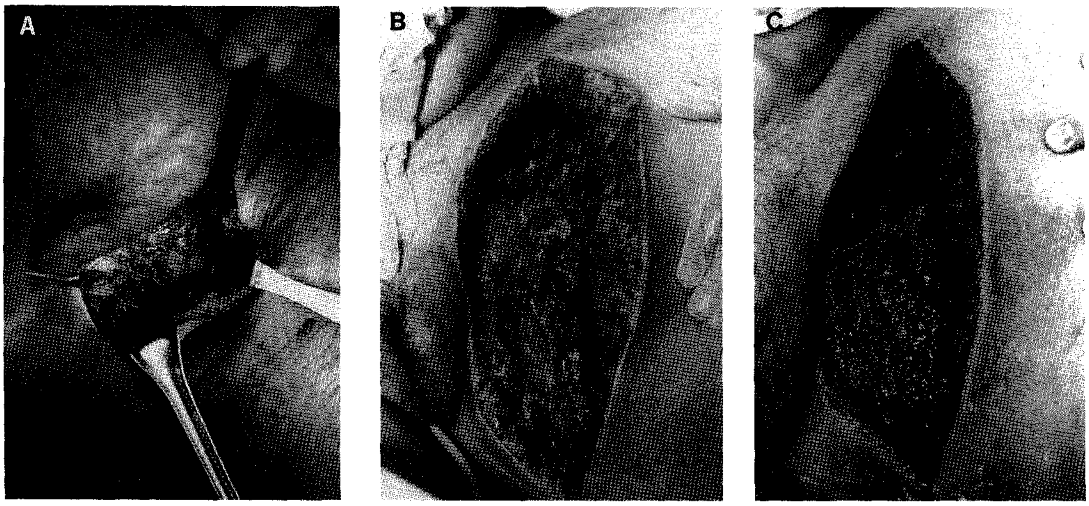

**Fig. 1.** Photographs of the vulvar area from case 1. **A:** Large area of involvement developed in 24 hours and was extensively débrided. **B:** Continued tissue necrosis and loss of resistance to probing was noted at 36 hours and required repeat excision. **C:** Granulation tissue and healing of wound bed 14 days after original surgery.

ing complaint. The abscess was needle aspirated, and the patient was sent home on oral dicloxacillin 500 mg q6h.

The following day, she returned to the emergency room complaining of increased pain and redness in the left groin area and subjective fever. Exam was significant for a temperature of 38°C, blood pressure 138/78, heart rate 88, respiratory rate 20. The left vulva was slightly edematous and erythematous. The left groin had an 8 × 10 cm erythematous area that extended down the inner thigh and was warm and tender to the touch. She had no clubbing or cyanosis of her extremities, nor did she have any pelvic masses or tenderness. She was alert and oriented and had a normal neurological exam.

She was admitted to the hospital and placed on intravenous ampicillin (2 mg IV q4h), gentamicin (1 mg/kg IV q8h), and clindamycin (900 mg IV q8h). Laboratory evaluation revealed an elevated white blood cell count of $17{,}400/mm^3$ and a glucose of 168 mg/dl. The next day the area of inflammation had extended to 20 cm down the left inner thigh, and foul-smelling purulent material exuded from the aspirated lesion. The patient was taken to the operating room where exploration of the groin area revealed multiple loculated abscess cavities extending from the left groin superior to the inguinal ligament and inferior to the femoral triangle and containing foul-smelling yellow-greenish pus. This material was sent for gram stain and aerobic and anaerobic cultures. Blood was sent for *Clostridium* toxin and aerobic and anaerobic cultures. The involved area was dissected underneath the subcutaneous tissue to the inferior margin of the left vulva (Fig. 1A). The necrotic tissue was excised to the point of bleeding with no loss of resistance to probing, and the area was irrigated with 6 liters of saline and packed.

Post-operatively, she was managed with subcutaneous insulin to maintain tight glucose control. Deep vein thrombosis (DVT) prophylaxis with a pneumatic compression hose on both legs and subcutaneous heparin 5,000 units twice daily was initiated in the operating room and continued post-operatively. Wound debridement and cleaning with one-quarter strength Dakin's solution (163 ml sodium hypochlorite 5.25% plus $0.325_g$ sodium bicarbonate in 3,637 ml water) were performed twice daily, and sterile gauze was used for packing the wound. After 36 hours, further necrosis of the wound edges was apparent. She was again taken to surgery, where blunt probing revealed loss of tissue resistance superiorlaterally close to the margin of the inguinal ligaments as well as medially past the inferior margin of the vulva and inner thigh (Fig. 1B). The skin, superficial fascia, and subcutaneous tissue of the areas were excised to the point of bleeding and no loss of resistance to probing.

Hemostasis was attained, and the wound was packed with sterile gauze.

Ampicillin, clindamycin, and gentamicin were continued, as were sliding scale insulin, DVT prophylaxis, and twice daily wound débridement and dressing changes. Aztreonam (2 g IV q6h) was substituted to reduce the risk of renal toxicity, which was significant in this patient. When no further signs of infection were present in the wound bed, one-quarter strength Dakin's solution was deleted, and sterile saline was substituted for wound care. The wound was cleaned with Biolex spray (a dilute aloe vera solution) and gel to promote granulation. Antibiotics were stopped after 10 additional days, after she had been afebrile for 48 hours. By this time, the wound was granulating well (Fig. 1C).

Aerobic cultures demonstrated both gram-negative (*E. coli, Klebsiella pneumoniae*) and gram-positive rods (*Corynebacterium* sp). Anaerobic cultures showed gram-negative rods (*Bacteroides* sp). Blood cultures and *Clostridium* toxin assay were negative. Pathological examination revealed necrotic skin with abscess and underlying fat necrosis. The patient was discharged home on hospital day 25 on twice daily dressing changes, and was followed with weekly visits for 6 weeks. She was doing well at her 6-month follow-up.

### Case 2

A 65 year old black woman ($G_7P_5A_2$) was admitted to Parkland Memorial Hospital for a hysterectomy after fractional dilatation and curettage revealed dysfunctional endometrium with cellular atypia. Because of her significant medical problems including hypertension, coronary artery disease, non-insulin dependent diabetes mellitus, degenerative joint disease, and a history of a mild stroke, she was evaluated by the medicine department consult staff and cleared for surgery. She had a 30 pack/year history of smoking and denied alcohol use. Physical examination revealed an obese (235 pounds) woman, blood pressure 150/90, heart rate 90, respiratory rate 20, temperature 37°C. She underwent a total abdominal hysterectomy, bilateral salpingo-oophorectomy, and appendectomy without complication. Pathology revealed multiple leiomyomata and no gross endometrial tumor.

On post-operative day 1 (POD 1), she had a maximum temperature of 38.5°C. Her incision was draining a small amount of serosanguineous fluid but did not look infected. On POD 2 she had a maximum temperature of 38.5°C, which was attributed to pulmonary atelectasis. She quickly defervesced and remained afebrile. On POD 4, a slight serosanguineous discharge was noted with wound separation apparent on probing. Her staples were removed to allow drainage, and the wound was packed with saline-moistened sterile gauze covered with dry sterile dressing and changed twice daily.

She remained afebrile until POD 6, when her temperature spiked to 38.4°C. At this time, her bandages contained greenish purulence, and the wound edges were necrotic and foul-smelling. When the wound was probed, loss of resistance of the subcutaneous tissue superficial to the rectal fascia extended 6 cm bilaterally. Only minimal skin erythema was present, but the wound margins were anesthetic. Importantly, the patient maintained normal bowel function, and her fluid balance and electrolytes were well controlled. Necrotizing fasciitis was suspected, and the patient was placed on IV ampicillin (2 g IV q4h), gentamicin (1 mg/kg IV q8h), and clindamycin (900 mg IV q8h) and taken to the operating room that same day. The areas of necrosis were excised to the point at which no loss of resistance was encountered and brisk capillary bleeding was evident. Tissue was sent for aerobic and anaerobic cultures. The wound was irrigated with 3 liters of saline and packed with sterile gauze.

Post-operatively, she was maintained with careful fluid management, and her triple antibiotics were continued. The wound was debrided and cleaned with one-quarter strength Dakin's solutin twice daily. The patient defervesced after 6 days, and granulation tissue became apparent. Hydrotherapy in a whirlpool bath was begun at this time. The wound occasionally required sharp débridement of necrotic debris, but otherwise it continued to granulate well. The wound culture revealed *Proteus mirabilis*. Pathology showed marked acute and chronic inflammation, necrosis, ulceration, and organizing fat necrosis. The patient was discharged 27 days after her admission on twice daily dressing changes and weekly follow-up in clinic for 6 weeks. She was doing well 4 months post-operatively.

### DISCUSSION

One of the earliest descriptions of hospital gangrene was written in 1871 by Joseph Jones, a Con-

**TABLE 1. Risk factors for necrotizing fasciitis**

| |
|---|
| Age over 50[a] |
| Arteriosclerosis[a] |
| Diabetes[a] |
| Obesity |
| Smoking |
| Previous radiation |
| Operative trauma |

[a]Major risk factors.

federate Army surgeon: "a purple or blue spot is first perceived that is sometimes raised, and contains serum below. The skin in the affected spot may melt away in 24 hours, whilst a deep blue and purple, almost black, areola surrounding the dead mass, spreads rapidly in an ever increasing circle. This is witnessed most generally in the worst and fatal cases."[4] The first large series was published by Meleney in 1924.[5] He studied 20 cases in China from which he cultured hemolytic streptococci and hence called the disease hemolytic streptococcal gangrene. The term *necrotizing fasciitis* was coined in 1952 by Wilson.[6] The first vulvar cases were described in 1972 by Roberts.[7]

The exact incidence of necrotizing fasciitis is not known, but fortunately it is uncommon. It has been described in all ages and in almost every site of the body. It is most commonly found in the lower extremities, followed by the upper extremities, abdomen, perineum, groin, back, and buttocks.[28] The initiating event is usually a minor penetrating injury and involves a surgical site less than half the time. In Rea's series of 44 patients with necrotizing fasciitis, 80% of the cases originated outside the hospital with minor injuries such as abrasions, cuts, bruises, boils, and insect bites, and only 20% were post-surgical infections.[2] In eight of the cases, no specific initiating factor could be found.

Those at highest risk of necrotizing fasciitis are those whose repair mechanisms are compromised due to peripheral vascular damage. The most common risk factors are summarizd in Table 1. The most frequent predisposing risk factors are advanced age followed closely by arteriosclerosis and diabetes. Rea first noticed the association of these factors with necrotizing fasciitis in 1970.[2] Of 44 cases studied, 45.5% were over age 50, 22.2% had arteriosclerosis, and 18.2% had diabetes.[2] In Roberts and Hester's and Roberts's collective cases of 22 patients with necrotizing fasciitis of the vulva, 68.1% were over age 50, and all but one, or 95.5%, were diabetic.[7,9] He did not comment on patients with arteriosclerosis. Other factors thought to predispose to necrotizing fasciitis are hypertension, vascular disease, obesity, renal failure, immunosuppression, a history of radiation therapy, and operative trauma.[10,11]

Necrotizing fasciitis is often misdiagnosed as cellulitis or a simple abscess because of the misleading symptoms and signs of its victims. Early skin changes include erythema, tenderness, and edema extending beyond the area of erythema.[1] These are usually accompanied by fever, malaise, and other systemic toxic signs. Early on, the skin may be intact.[11] Late signs include a purple or bluish discoloration, vesicles filled with red-black fluid, crepitance, cutaneous anesthesia, and necrosis. Once these late signs appear, however, the area of underlying destruction is usually large, and severe systemic toxicity develops.

The pathognomonic sign present in 100% of the cases is subcutaneous and superficial fascial necrosis.[2] Classically, this extends along fascial planes beyond the area of skin involvement. When blunt dissection with a probe or even a finger is carried out, the superficial tissue is sheared away without resistance. Wilson admonished that when undermining of this nature is demonstrated, the patient should receive immediate surgical therapy.[6]

The bacterial etiology of necrotizing fasciitis is polymicrobial (Table 2). In Meleney's series of 20 in 1924, the predominant organism was the hemolytic streptococcus, which was present in all cases.[5] Staphylococci was found in only 10%. Wilson, in contrast, reported in 1952 that 88% of his cases were infected with staphylococci.[6] Rea showed that streptococci and staphylococci were present in equal proportions, representing 43% each among his cases.[2] The majority of his cases grew out more than one organism, however. In a recent series by Stephenson the organisms most currently found were anaerobic *Peptostreptococcus* and *Bacteroides*.[12] These differences are most likely attributed to the improved techniques for culturing anaerobic organisms in recent years. Other bacteria reported to be involved include *E. coli, K. pneumoniae, Enterobacter, Peptococcus,* and *Pseudomonas*.[11] *Vibrio* sp. can be seen in wounds exposed to sea water.[13] *Clostridium* sp. are found less commonly in necrotizing fasciitis, even in those cases with subcutane-

**TABLE 2. Polymicrobial etiology of necrotizing fasciitis**

| Aerobes | Anaerobes |
|---|---|
| Gram-positive | |
| Cocci | |
| Group A, B, D streptococci[a] | *Peptostreptococcus* sp.[a] |
| Other α- and γ-streptococci | *Peptococcus* sp.[a] |
| *Staphylococcus aureus*[a] | |
| *Staphylococcus epidermidis* | |
| Rods | |
| Lactobacilli | *Clostridium* sp. |
| Diphtheroids | *Propionibacterium* sp. |
| | *Eubacterium* sp. |
| Gram-negative | |
| Cocci | |
| *Neisseria gonorrhoeae* | *Veillonella* sp. |
| Rods | |
| *E. coli*[a] | *Bacteroides fragilis* group[a] |
| *Klebsiella pneumoniae*[a] | Other *Bacteroides* sp. |
| *Enterobacter* sp.[a] | *Fusobacterium* sp. |
| *P. mirabilis* | |
| *Pseudomonas aeruginosa*[a] | |

[a]Most common isolates.

**TABLE 3. Diagnostic clues for necrotizing fasciitis**

- Cellulitis that fails to respond to antibiotic therapy
- Edema beyond the area of erythema
- Development of ecchymosis or vesicles over an area of cellulitis
- Presence of gas in the tissue as demonstrated by palpation (crepitus)

ous emphysema. Subcutaneous emphysema is thought to occur by aerobic and anaerobic bacteria synergistically forming hydrogen and nitrogen gas.[1] Gas formation is mostly associated with *E. coli*, microaerophilic *Streptococcus*, and *Bacteroides* sp.[13]

Diagnosis of necrotizing fasciitis can be difficult and is often made after the disease is widespread. A high clinical suspicion must be maintained to enable early and accurate diagnosis. Important diagnostic clues that lead to high suspicion for necrotizing fasciitis are listed in Table 3. Fisher emphasized the importance of radiological studies to demonstrate soft-tissue gas.[14] He showed that while crepitus was found in 29% of patients with necrotizing fasciitis, soft-tissue gas was found by x-ray in 100%. Fisher also gave six clinical criteria to satisfy the diagnosis of necrotizing fasciitis: 1) extensive necrosis of supeficial fascia with widespread undermining of surrounding tissue; 2) moderate to severe systemic toxic reaction with altered mental status; 3) absence of muscle involvement (vs. the prominent myonecrosis seen in certain clostridial infections and synergistic necrotizing cellulitis); 4) failure to demonstrate clostridia in wound and blood cultures; 5) absence of major vascular occlusion; and 6) pathological examination of débrided tissue showing intense leukocyte infiltration, focal necrosis of fascia and surrounding tissue, and thrombosis of the microvasculature.[14] Although clostridial species are typically not present in necrotizing fasciitis, it is important to rule out the presence of this pathogen in the wound, as this may mean the deep fascia and muscle are involved (clostridial myonecrosis). At surgery, a frozen section can aid in this diagnosis. Classically, necrotizing fasciitis shows necrosis of the superficial fascia and subcutaneous tissue with an intense polymorphonuclear infiltration and presence of multiple microorganisms on gram stain.[11,13] A clostridial infection will be remarkable for the absence of an inflammatory infiltrate and the presence of many gram-positive rods.[13] If the latter is the case, close inspection of the deep fascia and underlying muscle is warranted. Stephenson et al.[12] reported the presence of *Clostridium tetani* in one patient with necrotizing fasciitis of the vulva but indicated that no myonecrosis was present, as has been reported in cases with *Clostridium perfringens*.

Once suspicion is high for necrotizing fasciitis, the patient should be taken to the operating room for exploration and surgical débridement. Brewer and Meleney are credited with the first two successful surgical treatments of necrotizing fasciitis, then called progressive gangrenous infection of the skin and subcutaneous tissues, which occurred in and around abdominal incisions for operative care of acute perforative appendicitis.[15] In one case, an incision was made circumferentially around the infected area, down to the deep fascia, and the wound was packed. The enclosed area sloughed, but there was no progression of disease, so the wound ultimately granulated closed. The other case was successfully treated with excision of involved tissue to viable margins.

If inspection shows the characteristic skin ecchymosis, it is likely that the area of undermining is great. It is important, however, to incise and débride the entire extent of disease, until there is no further loss of resistance to blunt probing and until the tissue bleeds easily when cut. As Wilson stated, "to postpone surgery and use massive doses of antibiotics is ineffective and, in addition, the incision which must eventually be made must then be more extensive in a sicker patient."[6] In such a patient, the post-operative care should be in an intensive care unit in isolation, much like that of a burn victim. Insensible fluid losses will be great, and the prospect of hypovolemia with hemoconcentration is high.[13] Aggressive fluid and electrolyte management is important, along with periodic blood transfusions to correct anemia due to red cell destruction. Broad spectrum antibiotics (i.e., ampicillin, gentamicin, clindamycin) should be used until the identity and sensitivities of pathogens are known. We have found twice daily dressing changes and wound débridement to be sufficient for wound care. The wound is cleaned in this manner with 3% hydrogen peroxide or one-quarter strength Dakin's solution, and packed with sterile gauze. When the old dressings no longer have a foul odor, saline can be substituted for one-quarter strength Dakin's solution for wound cleaning. We also use Biolex gel (an aloe vera-containing gel) to promote wound healing.

Hyperbaric oxygen treatments have been cited as reducing edema, halting progression of tissue necrosis, and decreasing mortality, especially among wounds infected with clostridia.[13,16] Split-thickness skin grafting is an option for re-epithelializing large areas after adequate granulation. Fortunately, in our cases the extent of the disease was not so severe as to require disfiguring surgery, and the wounds were able to granulate in completely.

Mortality rates for necrotizing fasciitis are generally quoted as 30–60%,[8] and antibiotics have not significantly changed this.[2] Death is usually immediately attributed to overwhelming sepsis, complications of diabetes (ketoacidosis), vascular insufficiency, and hemodynamic collapse.[3,8] Not only do old age and diabetes seem to be the most important predisposing factors for necrotizing fasciitis, but they are also the two leading factors for a grave prognosis.[3] Rea showed the mortality rate in patients over 50 years of age to be 67%, while that in diabetics to be 63%.[2] Roberts and Hester and Roberts had a mortality rate from his cases of 47% and 38%, respectively.[7,9] Stephenson reported a 62% mortality in patients over age 50 and a 55% mortality rate in diabetic patients.[12] Significantly, all of the fatalities had one or both of these significant high-risk factors. The most important factors for survival are rapid diagnosis, and rapid and adequate surgical treatment. Rea once again stresses this fact by showing that the average time from onset of disease to diagnosis and treatment of those who lived was 4 days, while that of those who died was 7 days.[2] Stephenson found that 48 hours was a more significant time frame, after which the mortality rate was 75%.[12] The mortality rate in Wilson's collection was only 8.7%; this low number may be attributed in part to the expertise of the house officers in the teaching program at Parkland Memorial Hospital, who had been trained to recognize the clinical manifestations of the disease.[6]

### SUMMARY

We have presented two cases of necrotizing fasciitis recently managed at a large Ob/Gyn residency training program. We have reviewed the patients' histories, risk factors, signs and symptoms, bacteriology, pathology, therapy, and mortality factors associated with this life-threatening disease process. Our patients were both in a high mortality risk category for necrotizing fasciitis in that both were over 50 years old, had diabetes, and were obese. One patient had documented arteriosclerosis, and the other had risk factors for it (obesity, smoking, and diabetes). Despite these high risk factors, both of these patients did well. We agree with Wilson that high suspicion and prompt action on the part of the house officers involved in the care of these women were the keys to their successful outcome.[6] Some of the general guidelines for the management of necrotizing fasciitis at Parkland Memorial Hospital are listed in Table 4. In the first case, the main clinical clue was the failure of the presumed cellulitis to respond to antibiotics. In the second, it was the loss of tissue resistance to blunt probing. Both were taken to surgery within 3 days of the presenting problem.

The first case showed the typical polymicrobial nature of necrotizing fasciitis, whereas in the second only one organism (*P. mirabilis*) was cultured. This may be due to the perioperative broad spectrum antibiotic prophylaxis given to this patient at the time of her hysterectomy.

Necrotizing fasciitis can occur in any surgical or nonsurgical insult. In the recent Ob/Gyn literature alone, there are cases reported from episiotomy, mini-laparotomy, diagnostic laparoscopy, and suprapubic catheter placement.[16–19] There are also reports of patients without an obvious original nidus of infection, or patients on standard treatment

**TABLE 4. Guidelines for managing necrotizing fasciitis at PMH**

1. Patients with suspicious lesions in the vulvar, groin, or thigh region with high-risk factors for necrotizing fasciitis are admitted for observation
2. Patients with presumed cellulitis who fail to respond to IV antibiotics are taken to surgery for wound exploration
3. Post-operative patients who have high-risk factors for necrotizing fasciitis and have wound separation with loss of tissue resistance are started on IV antibiotics and taken to the operating room for exploration
4. Junior house officers notify senior house officers and faculty experienced in the care of necrotizing fasciitis about any suspicious lesions or wound complications
5. Lectures on soft-tissue infections are included on a regular basis for residents and medical students
6. Wound care is managed by the same upper level resident on any service, and additional débridement and/or return to the operating room is performed as needed
7. Survival depends on early recognition and immediate surgical débridement to healthy tissue margins
   - Remove all indurated, edematous, crepitant tissue
   - Incise tissue to margins that bleed easily
   - Change wound dressings frequently
   - Initiate broad spectrum antibiotics regimens such as radiotherapy, chemotherapy, and anti-inflammatory drugs.[20–22]

It is often said that anyone who uses a certain kind of intervention or therapy should be prepared to deal with the consequences of that intervention or therapy. Among the surgical specialties, obstetricians and gynecologists frequently deal with heavily contaminated body areas; therefore, knowledge of surgical complications is imperative, especially one as life-threatening as necrotizing fasciitis.

### ACKNOWLEDGMENTS

The authors acknowledge the many hours of medical care provided to these patients by the following physicians: Deborah Lenart, M.D., Stacy Travis, M.D., and Michael Bevers, M.D. We appreciate the skillful manuscript preparation by Ms. Maria McFarland.

### REFERENCES

1. Shy KK, Eschenbach DA: Fatal perineal cellulitis from an episiotomy site. Obstet Gynecol 54:292–298, 1979.
2. Rea WJ, Wyrick WJ, Jr: Necrotizing fasciitis. Ann Surg 172:957–964, 1970.
3. Addison NA, Livengood CH, Hill GB, Sutton GP, Fortier KJ: Necrotizing fasciitis of vulvar origin in diabetic patients. Obstet Gynecol 63:473–479, 1984.
4. Jones J: Investigations upon the nature, causes, and treatment of hospital gangrene as it prevailed in the Confederate Armies 1861–65. New York, U.S. Sanitary Commission, Surgical Memories of the War of Rebellion, 1871. As quoted in Wilson, B: Necrotizing fasciitis. Am Surg 18:416–431, 1952.
5. Meleney FL: Hemolytic streptococcus gangrene. Arch Surg 9:317–364, 1924.
6. Wilson B: Necrotizing fasciitis. Am Surg 18:416–431, 1952.
7. Roberts DB, Hester LL, Jr: Progressive synergistic bacterial gangrene arising from abscesses of the vulva and Bartholin's gland duct. Am J Obstet Gynecol 114:285–289, 1972.
8. Lee RA: Postoperative necrotizing fasciitis. In Nichols DH, Anderson GW (eds): Clinical Problems, Injuries, and Complications of Gynecologic Surgery. Baltimore: Williams & Wilkins, pp 1–4, 1988.
9. Roberts DB: Necrotizing fasciitis of the vulva. Am J Obstet Gynecol. 157:568–571, 1987.
10. Livengood CH, Soper JT, Clarke-Pearson DL, Addison WA: Necrotizing fasciitis in irradiated tissue from diabetic women. J Reprod Med 36:455–458, 1991
11. Adelson MD, Joret DM, Gordon LP, Osborne NG: Recurrent necrotizing fasciitis of the vulva. J Reprod Med 36:818–822, 1991.
12. Stephenson H, Dotters DJ, Katz V, Droegmuller W: Necrotizing fasciitis of the vulva. Am J Obstet Gynecol 166:1324–1327, 1992.
13. Lewis RT: Soft tissue infections. In Wilmore DW (ed): Care of the Surgical Patient. New York: Scientific American, pp 6.1–6.15, 1988.
14. Fisher JR, Conway MJ, Takeshita RT, Sandoval MR: Necrotizing fasciitis: Importance of roentgenographic studies for soft-tissue gas. JAMA 241:803–806, 1979.
15. Brewer GE, Meleney FJ: Progressive gangrenous infection of the skin and subcutaneous tissues following operation for acute perforative appendicitis. Ann Surg 84:438–450, 1926.
16. Riseman JA, Zamboni WA, Curtis A, Graham DR, Konrad HR, Ross DS: Hyperbaric oxygen therapy for necrotizing fasciitis reduces mortality and the need for débridements. Surgery 108:847–850, 1990.
17. Sotrel G, Hirsch E, Edelin KC: Necrotizing fasciitis following diagnostic laparoscopy. Obstet Gynecol 62:675–695, 1983.
18. Bearman DM, Livengood CH, Addison WA: Necrotizing fasciitis arising from a suprapublic catheter site. J Reprod Med 33:411–413, 1988.
19. Cederma JP, Davies BW, Farkas SA, Sonta JA, Swورniowski T: Necrotizing fasciitis of the total abdominal wall after sterilization by partial salpingectomy. Am J Obstet Gynecol 162:138–139, 1990.
20. Fröhlich EP, Schein M: Necrotizing fasciitis arising from Bartholin's abscess. Isr J Med Sci 25:644–647, 1989.
21. Hoffman MS, Turnquist D: Necrotizing fasciitis of the vulva during chemotherapy. Obstet Gynecol 74:483–484, 1989.
22. Smith RJ, Berk SC: Necrotizing fasciitis and nonsteroidal anti-inflammatory drugs. South Med J 84:785–787, 1991.

## References

[PDF_00608] Addison W. A., Livengood C. H., Hill G. B., Sutton G. P., Fortier K. J. (1984). Necrotizing fasciitis of vulvar origin in diabetic patients.. Obstet Gynecol.

[PDF_00633] Adelson M. D., Joret D. M., Gordon L. P., Osborne N. G. (1991). Recurrent necrotizing fasciitis of the vulva. A case report.. J Reprod Med.

[PDF_00656] Bearman D. M., Livengood C. H., Addison W. A. (1988). Necrotizing fasciitis arising from a suprapubic catheter site. A case report.. J Reprod Med.

[PDF_00645] Brewer G. E., Meleney F. L. (1926). PROGRESSIVE GANGRENOUS INFECTION OF THE SKIN AND SUBCUTANEOUS TISSUES, FOLLOWING OPERATION FOR ACUTE PERFORATIVE APPENDICITIS: A STUDY IN SYMBIOSIS.. Ann Surg.

[PDF_00642] Fisher J. R., Conway M. J., Takeshita R. T., Sandoval M. R. (1979). Necrotizing fasciitis. Importance of roentgenographic studies for soft-tissue gas.. JAMA.

[PDF_00663] Fröhlich E. P., Schein M. (1989). Necrotizing fasciitis arising from Bartholin's abscess. Case report and review of the literature.. Isr J Med Sci.

[PDF_00665] Hoffman M. S., Turnquist D. (1989). Necrotizing fasciitis of the vulva during chemotherapy.. Obstet Gynecol.

[PDF_00630] Livengood C. H., Soper J. T., Clarke-Pearson D. L., Addison W. A. (1991). Necrotizing fasciitis in irradiated tissue from diabetic women. A report of two cases.. J Reprod Med.

[PDF_00659] Rappaport V. J., Hirata G., Yap H. K., Jordan S. C. (1990). Anti-vascular endothelial cell antibodies in severe preeclampsia.. Am J Obstet Gynecol.

[PDF_00606] Rea W. J., Wyrick W. J. (1970). Necrotizing fasciitis.. Ann Surg.

[PDF_00649] Riseman J. A., Zamboni W. A., Curtis A., Graham D. R., Konrad H. R., Ross D. S. (1990). Hyperbaric oxygen therapy for necrotizing fasciitis reduces mortality and the need for debridements.. Surgery.

[PDF_00620] Roberts D. B., Hester L. L. (1972). Progressive synergistic bacterial gangrene arising from abscesses of the vulva and Bartholin's gland duct.. Am J Obstet Gynecol.

[PDF_00628] Roberts D. B. (1987). Necrotizing fasciitis of the vulva.. Am J Obstet Gynecol.

[PDF_00604] Shy K. K., Eschenbach D. A. (1979). Fatal perineal cellulitis from an episiotomy site.. Obstet Gynecol.

[PDF_00668] Smith R. J., Berk S. L. (1991). Necrotizing fasciitis and nonsteroidal anti-inflammatory drugs.. South Med J.

[PDF_00636] Stephenson H., Dotters D. J., Katz V., Droegemueller W. (1992). Necrotizing fasciitis of the vulva.. Am J Obstet Gynecol.

[PDF_00615] WILSON B. (1952). Necrotizing fasciitis.. Am Surg.

[PDF_00619] WILSON B. (1952). Necrotizing fasciitis.. Am Surg.

